# Society Meetings

**Published:** 1906-05

**Authors:** 


### SOCIETY MEETINGS.

THE NATIONAL CONFEDERATION OF STATE MEDICAL EXAMINING AND LICENSING BOARDS will hold its Sixteenth Annual Meeting, in the State Capitol at Boston, Monday, June 4, 1906, at 2 p. m., under the presidency of Henry Beates, Jr., of Philadelphia. A number of interesting papers will be presented and questions of importance will be discussed.

An address will be delivered by Dr. Howard J. Rogers, First Assistant Commissioner of Education of the State of New York. A large representation from the several state boards is anticipated. Members who desire to present papers should communicate at once with the president, Dr. Henry Beates, Jr., 1504 Walnut St., Philadelphia, Pa.

---

THE AMERICAN ACADEMY OF MEDICINE will hold its thirty-first annual meeting at the Hotel Brunswick, Boston, Saturday and Monday, June 2 and 4, 1906, under the presidency of Donley C. Hawley, of Burlington, Vt. Communications relating to details of the meeting should be addressed to Dr. Charles McIntire, Secretary, Easton, Pa.

The National Association of United States Pension Examining Surgeons will hold its fifth annual meeting at the New American Hotel, Boston, June 4 and 5, 1906, under the presidency of Charles James Fox, of Hartford. A large meeting is anticipated and all who desire to present papers should correspond with the secretary, Dr. Porter Farley, of Rochester, N. Y.

The Buffalo Academy of Medicine held meetings during the month of April as follows:

*Section of Surgery.*—Tuesday, April 3, 1906 at 8:30 P. M. Program: Fibroid of the uterus complicated by pregnancy, with report of cases, Geo. Ben Johnston, Richmond, Va. Pressure fracture of the vertebræ, Bernard Bartow.

*Section of Medicine.*—Tuesday, April 10, 1906, at 8:30 P. M. Program: (a) Report of cases of aortic stenosis, Nelson G. Russell. (b) The diagnosis of insanity in borderline cases, Sidney A. Dunham. Discussion by Floyd S. Crego, James W. Putnam, and Arthur W. Hurd.

*Section of Obstetrics and Gynecology.*—Tuesday, April 24, 1906, at 8:30 P. M. Program: The extinction of the human unfit, Ely Van de Warker, Syracuse.

